# High carbohydrate antigen 19–9 levels indicate poor prognosis in patients with bladder cancer following radical cystectomy

**DOI:** 10.3389/fonc.2025.1550203

**Published:** 2025-08-18

**Authors:** Sang Won So, Jang Hee Han, Hyeong Dong Yuk, Chang Wook Jeong, Cheol Kwak, Ja Hyeon Ku, Seung-hwan Jeong

**Affiliations:** ^1^ Department of Urology, Seoul National University Hospital, Seoul, Republic of Korea; ^2^ Department of Urology, Seoul National University College of Medicine, Seoul, Republic of Korea

**Keywords:** bladder cancer, radical cystectomy, recurrence-free survival, prognosis, CA 19-9

## Abstract

**Introduction:**

Our team previously reported that elevated carbohydrate antigen (CA) 19–9 levels are associated with a worse prognosis in upper tract urothelial carcinoma (UTUC). Several studies have reported a correlation between high tumor burden and elevated CA19–9 levels in urothelial carcinomas. However, no studies have specifically examined the association between CA19–9 levels and outcomes of patients with bladder cancer who underwent radical cystectomy. Therefore, we aimed to evaluate the relationship of CA19–9 levels in bladder cancer patients following radical cystectomy.

**Materials and Methods:**

Among the 984 patients who underwent radical cystectomy at the Seoul National University Hospital between 1991 and 2022, 564 patients had available preoperative CA19–9 levels. The patients were divided into two groups: a low CA19–9 group (CA19-9 ≤ 37 U/mL) and high CA19–9 group (CA19-9 > 37 U/mL). Demographic parameters as well as preoperative and postoperative findings were compared between these two groups. Recurrence-free survival and overall survival were analyzed using multivariate Cox regression and Kaplan-Meier analyses.

**Results:**

Sex distribution, age, body mass index, and underlying diseases (hypertension and diabetes mellitus) were similar between the two groups. The clinical T and N stages were significantly higher in the high CA19–9 group (*P* = 0.028 and *P* = 0.019, respectively). The operative procedures, including open, laparoscopic, and robotic surgeries, were similarly performed in both groups. Pathologic T and N stages also tended to be higher in the high CA19–9 group (*P* < 0.001 and *P* = 0.005, respectively). In the multivariate Cox regression analysis, the recurrence risk in the high CA19–9 group was significantly higher than that in the low group (HR 1.646; 95% CI 0.070–2.533, *P* = 0.023). The 5-year recurrence-free survival rate was 53.5% and 35.5% in the low and high CA19–9 groups, respectively (*P* < 0.001). Overall survival tended to be worse in the high CA19–9 group; however, this difference was not statistically significant.

**Conclusions:**

A high CA19–9 level is associated with a higher tumor burden in patients with bladder cancer. Furthermore, high CA19–9 levels are correlated to higher pathologic T and N stages after radical cystectomy and worse recurrence-free survival.

## Introduction

Bladder cancer is the 10^th^ most common cancer worldwide, and the prevalence in men is four times higher than that in women (1). Radical cystectomy is considered for curative purposes in cases of muscle-invasive bladder cancer (MIBC) classified as T2 or higher, without distant metastasis (2). Early radical cystectomy is also an option for some patients including cases classified lower than T2, especially when BCG treatment fails or the patient has high-risk non-MIBC with a specific histologic subtype (3). Depending on the stage and patient condition, neoadjuvant chemotherapy such as GC (Gemcitabine, Cisplatin) or MVAC (Methotrexate, Vinblastine, Doxorubicin, Cisplatin) may be administered before radical cystectomy (4). Currently, the diagnosis of bladder cancer is made using cystoscopy, urine cytology, and computed tomography imaging for patients with symptoms such as painless gross hematuria (5). However, cystoscopy is an invasive procedure, causing pain and discomfort during the procedure. Urine cytology is relatively non-invasive but usually interpreted with other diagnostic tools due to its low sensitivity (6). Alternatively, serum diagnostic markers may simplify diagnosis procedure with a relatively small blood sample. Carcinoembryonic antigen (CEA), carbohydrate antigen (CA19-9), and cytokeratin 19 fragment (CYFRA21-1) are currently being considered as potential markers for diagnosing and evaluating bladder cancer, although none have been definitively established (7). Among these markers, CA19–9 has been previously used as a diagnostic and prognostic biomarker in gastrointestinal tumors, such as colorectal and pancreatic cancers (8, 9). In our previous study, we showed a correlation between CA19–9 levels and tumor burden as well as prognosis in upper tract urothelial carcinoma (UTUC) (10). Moreover, elevated CA19–9 is associated with high tumor burden and poor prognosis in urothelial cancer (11, 12), and may also be related to the prognosis in bladder cancer (13). However, no studies have investigated the relationship between CA19–9 and postoperative survival in patients with bladder cancer with high risk or high tumor burden who underwent radical cystectomy. In this study, we aimed to determine the relationship between CA19–9 levels and several diagnostic/prognostic factors in patients with bladder cancer who underwent radical cystectomy.

## Materials and methods

### Study population

This study included 984 patients with bladder cancer who underwent radical cystectomy at the Seoul National University Hospital (SNUH) from 1991 to 2022. The study was conducted with approval from the SNUH Institutional Review Board (IRB No. 2411-075-1587) (14). Among the 984 patients, the study included patients whose preoperative serum CA 19–9 levels were measured. Patients in which treatment was being administered for gastrointestinal malignant tumors, such as pancreatic cancer or rectal cancer, were excluded.

### Study design

A retrospective cohort study was conducted. Patients were divided into two groups based on their serum CA 19–9 levels. For the measurement of CA19-9, peripheral venous blood 3ml from each patient was collected in a serum separate tube before surgery. A chemiluminescent microparticle immunoassay was used for measurement and all test results were measured using the same method on the day of blood collection, in single laboratory of SNUH. Considering that the normal level of serum CA 19–9 is below 37 U/mL, the patients were divided into a low CA 19–9 group (≤ 37 U/mL) and high CA 19–9 group (> 37 U/mL) (15).

Basic information and characteristics of patients were also included such as gender, age, and presence of underlying diseases (e.g., hypertension, diabetes mellitus). Preoperative factors such as whether neoadjuvant chemotherapy was administered and clinical stage (T stage, N stage) were analyzed. Postoperative factors included the pathologic stage (T stage, N stage) and surgical methods (open, laparoscopic, robotic).

### Statistical analysis

The patient’s clinical information, including age and body mass index, was analyzed using a two-tailed t-test to calculate the median values. Several pre- and post-operative factors, such as gender, underlying diseases (hypertension, diabetes mellitus), clinical stage (T stage, N stage), pathologic stage (T stage, N stage), neoadjuvant chemotherapy status, and surgical methods (open, laparoscopic, robotic), were analyzed using the chi-square test. Recurrence-free survival and overall survival were analyzed using multivariate Cox regression and Kaplan-Meier analyses. Data analysis was performed using The XLSTAT Life Science. Statistical significance was set at a *P*-value of less than 0.05.

## Results

### Patient characteristics

Among the 564 patients who underwent radical cystectomy with available preoperative serum CA 19–9 levels, 514 patients were in the low CA19–9 group and 50 patients were in the high CA19–9 group. In both groups, there were no significant differences in gender (male proportion 82.1% [422] *vs* 72.0% [36], *p* = 0.081), average age (67.6 *vs* 67.0 years, *p* = 0.710), and body mass index (23.9 *vs* 23.6, *p* = 0.553) (**Table 1**). There was also no significant difference in the prevalence of underlying diseases such as hypertension (49.8% [256] *vs* 50.0% [25], *p* = 0.979) and diabetes (26.7% [137] *vs* 32.0% [16], *p* = 0.417) between the two groups.

**Table 1:** Patient characteristics (*n*=564).

Characteristics	CA19–9 Low (n=514)	CA19–9 High (n=50)	*P*-value
Sex			
Male (n,%)	422 (82.1)	36 (72.0)	0.081
Female (n,%)	92 (17.9)	14 (28.0)	
Age	67.6	67.0	0.710
BMI	23.9	23.6	0.553
HTN (n,%)	256 (49.8)	25 (50.0)	0.979
DM (n,%)	137 (26.7)	16 (32.0)	0.417

Values are presented as numbers (%) or mean. BMI, body mass index; DM, diabetes mellitus; HTN, hypertension.

### Treatment option

The administration of neoadjuvant chemotherapy and surgical methods (open, laparoscopic, robotic) performed on patients with bladder cancer were analyzed in this study (**Table 2**). The rates of neoadjuvant chemotherapy administered were 25.6% [131] and 32.0% [16] in the low and high CA19–9 groups, respectively, and the difference was not statistically significant (*p* = 0.317). Regarding the surgical methods, open method was used in 58.2% [299] versus 60.0% [30], laparoscopy in 0.8% [4] versus 0% [0], and robotic surgery in 41.1% [211] versus 40.0% [20] in the low and high CA 19–9 groups, respectively, with no significant difference observed (*p* = 0.808).

**Table 2:** Disease characteristics of patients (*n*=564) based on CA19–9 levels.

Characteristics	CA19–9 Low (*n*=514)	CA19–9 High (*n*=50)	*P*-value
Clinical T stage			
CIS (*n*,%)	14 (2.7)	1 (2.0)	0.028
Ta (*n*,%)	18 (3.5)	1 (2.0)	
T1 (*n*,%)	133 (25.9)	9 (18.0)	
T2 (*n*,%)	237 (46.1)	17 (34.0)	
T3 (*n*,%)	73 (14.2)	15 (30.0)	
T4 (*n*,%)	39 (7.6)	7 (14.0)	
Clinical N stage			
N0 (*n*,%)	438 (85.2)	34 (68.0)	0.019
N1 (*n*,%)	32 (6.2)	7 (14.0)	
N2 (*n*,%)	40 (7.8)	8 (16.0)	
N3 (*n*,%)	4 (0.8)	1 (2.0)	
Neoadjuvant chemotherapy status			
Neoadjuvant CTx (*n*,%)	131 (25.6)	16 (32.0)	0.317
Operation			
Open	299 (58.2)	30 (60.0)	0.808
Laparoscopic	4 (0.8)	0	
Robotic	211 (41.1)	20 (40.0)	
Pathologic T stage			
T0 (*n*,%)	109 (21.2)	6 (12.0)	<0.001
CIS (*n*,%)	50 (9.7)	3 (6.0)	
Ta (*n*,%)	27 (5.3)	3 (6.0)	
T1 (*n*,%)	89 (17.3)	4 (8.0)	
T2 (*n*,%)	76 (14.8)	5 (10.0)	
T3 (*n*,%)	124 (24.1)	15 (30.0)	
T4 (*n*,%)	39 (7.6)	14 (28.0)	
Pathlogic N stage			
N0 (*n*,%)	390 (75.9)	27 (54.0)	0.005
N1 (*n*,%)	34 (6.6)	5 (10.0)	
N2 (*n*,%)	52 (10.1)	13 (26.0)	
N3 *(n*,%)	3 (0.6)	1 (2.0)	
Nx (*n*,%)	35 (6.8)	4 (8.0)	

Values are presented as numbers (%) or median. CTx, Chemotherapy; CIS, Carcinoma *in situ*.

### Pathologic results

The distribution of clinical stage (T stage, N stage) and postoperative pathologic stage (T stage, N stage) were compared and analyzed for each group (**Table 2**). When comparing the clinical T stage, the low CA19–9 group had CIS 2.7%, Ta 3.5%, T1 25.9%, T2 46.1%, T3 14.2%, and T4 7.6% and the high CA19–9 group presented CIS 2.0%, Ta 2.0%, T1 18.0%, T2 34.0%, T3 30.0%, and T4 14.0% (*p* = 0.028). Regarding the clinical N stage, the low CA19–9 group had N0 85.2%, N1 6.2%, N2 7.8%, and N3 0.8%, while the high CA19–9 group showed N0 68.0%, N1 14.0%, N2 16.0%, and N3 2.0% (*p* = 0.019). Overall, the high CA19–9 group had significantly more advanced clinical T and N stages compared to those of the low CA19–9 group.

Subsequently, when comparing the pathologic T stage, the low CA19–9 group had T0 21.2%, CIS 9.7%, Ta 5.3%, T1 17.3%, T2 14.8%, T3 24.1%, and T4 7.6%, whereas the high CA19–9 group had T0 12.0%, CIS 6.0%, Ta 6.0%, T1 8.0%, T2 10.0%, T3 30.0%, and T4 28.0% (*p* < 0.001). Regarding the pathologic N stage, in the low CA19–9 group, the distribution was N0 75.9%, N1 6.6%, N2 10.1%, N3 0.6%, and Nx 6.8%, while in the high CA19–9 group, it was N0 54.0%, N1 10.0%, N2 26.0%, N3 2.0%, and Nx 8.0% (*p* = 0.005). Overall, the high CA19–9 group had significantly more advanced pathologic T and N stages compared to those of the low CA19–9 group.

### Recurrence-free survival and overall survival

For each group, recurrence-free survival and overall survival were compared using multivariate Cox regression analysis. First, a Cox proportional hazards model for cancer recurrence was calculated (**Table 3**). The hazard ratio (HR) for age was 1.002 (95% CI 0.988–1.017, *p* = 0.753), indicating no significant effect on recurrence. Conversely, CA 19–9 levels showed a significant effect on recurrence as the hazard ratio of the high CA 19–9 group was 1.646 (95% CI 0.070–2.533, *p* = 0.023) compared to that of the low CA 19–9 group. In terms of the pathologic T stage, the hazard ratio for recurrence significantly increased in all stages T1 and above compared to that of T0. The hazard ratio was 2.503 (95% CI 1.280–4.896, *p* = 0.007) for T1, 3.217 (95% CI 1.667–6.206, *p* < 0.001) for T2, 5.298 (95% CI 2.892–9.704, *p* < 0.001) for T3, and 8.715 (95% CI 4.470–16.993, *p* < 0.001) for T4. Concerning the pathologic N stage, the risk of recurrence was higher in all stages compared to that of N0: HR 2.627 (95% CI 1.671–3.340, *p* < 0.001) for N1, 2.216 (95% CI 1.470–3.340, *p* < 0.001) for N2, and HR 2.639 (95% CI 0.815–8.548, *p* = 0.007) for N3. The Cox proportional hazards model for overall survival was subsequently calculated (**Table 4**). The hazard ratio for age was 1.025 (95% CI 1.008–1.042, *p* = 0.004), indicating a significant impact on overall survival. In contrast, the high CA 19–9 group showed a higher risk compared to that of the low group (HR 1.079; 95% CI 0.657–1.774, *p* = 0.763); however, the difference was not statistically significant. Regarding pathologic T stage, hazard ratios for overall survival significantly increased at stage T1 and above, with HR 2.011 (95% CI 1.020–3.967, *p* = 0.044), HR 2.154 (95% CI 1.115–4.159, *p* = 0.022), HR 4.242 (95% CI 2.343–7.677, *p* < 0.001), and HR 5.713 (95% CI 1.115–4.159, *p* < 0.001) for T1, T2, T3, and T4, respectively. Regarding the pathologic N stage, there was a significant increase in risk for overall survival in the N2 stage, with HR 1.987 (95% CI 1.295–3.050, *p* = 0.002).

**Table 3:** Cox regression analysis for recurrence.

Characteristics	HR (95% CI)	*P*-value
Age	1.002 (0.988 – 1. 017)	0.753
CA 19-9		
Low	Reference	Reference
High	1.646 (0.070 – 2.533)	0.023
Pathologic T stage		
T0	Reference	Reference
CIS	1.056 (0.399 – 2.794)	0.912
Ta	1.546 (0.502 – 4.759)	0.447
T1	2.503 (1.280 – 4.896)	0.007
T2	3.217 (1.667 – 6.206)	0.0005
T3	5.298 (2.892 – 9.704)	< 0.001
T4	8.715 (4.470 – 16.993)	< 0.001
Pathologic N stage		
N0	Reference	Reference
N1	2.627 (1.671 – 4.129)	< 0.001
N2	2.216 (1.470 – 3.340)	< 0.001
N3	2.639 (0.815 – 8.548)	0.007
Nx	2.083 (1.226 – 3.542)	0.023

**Table 4:** Cox regression analysis for overall survival.

	HR (95% CI)	*P*-value
Age	1.025 (1.008 – 1.042)	0.004
CA 19-9		
Low	Reference	Reference
High	1.079 (0.657 – 1.774)	0.763
Pathologic T stage		
T0	Reference	Reference
CIS	1.210 (0.521 – 2.811)	0.657
Ta	1.594 (0.521 – 4.871)	0.414
T1	2.011 (1.020 – 3.967)	0.044
T2	2.154 (1.115 – 4.159)	0.022
T3	4.242 (2.343 – 7.677)	< 0.0001
T4	5.713 (2.856 – 11.430)	< 0.0001
Pathologic N stage		
N0	Reference	Reference
N1	1.148 (0.647 – 2.037)	0.637
N2	1.987 (1.295 – 3.050)	0.002
N3	1.525 (0.466 – 4.992)	0.486
Nx	1.361 (0.743 – 2.493)	0.318

**Figure 1:**
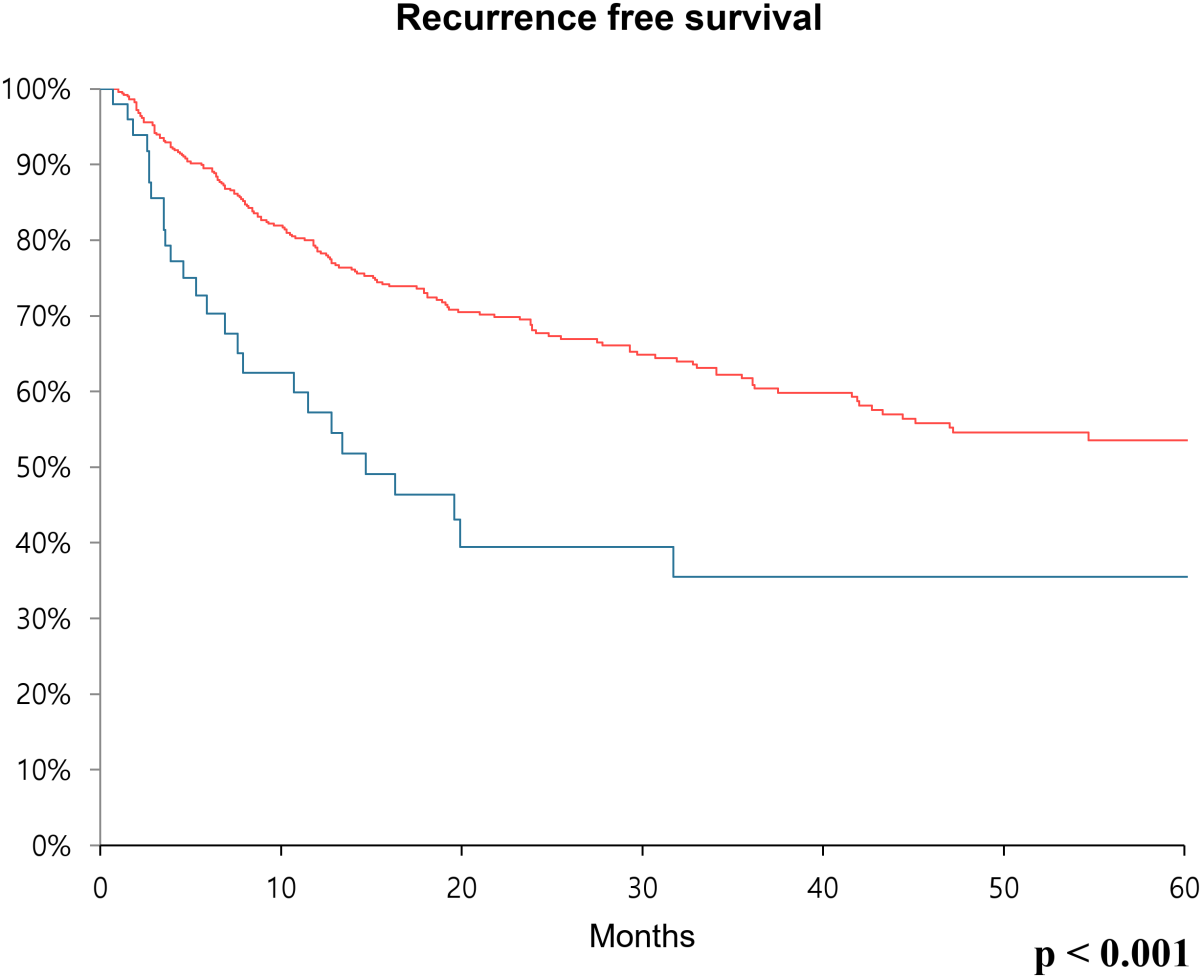
Kaplan-Meier survival curve over 60 months showing recurrence-free survival rates comparing the low CA19-9 group (red line) and high CA19-9 group (blue line) after radical cystectomy.

**Figure 2:**
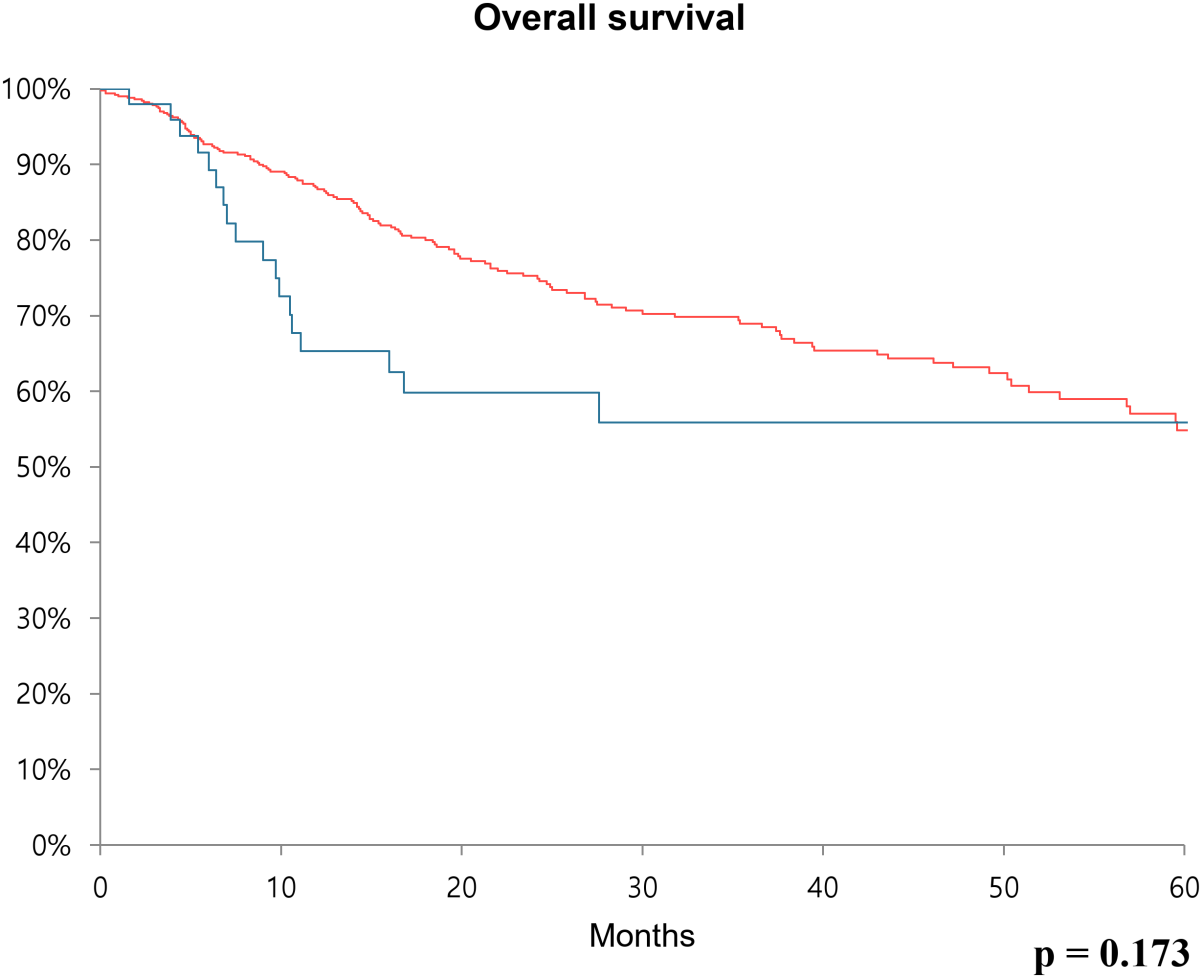
Kaplan-Meier survival curve over 60 months showing overall survival rate comparing the low CA19-9 group (red line) and high CA19-9 group (blue line) after radical cystectomy.

Furthermore, recurrence-free survival and overall survival were calculated using Kaplan-Meier analysis. In the case of 5-year recurrence-free survival, the low CA19–9 group had a survival rate of 53.5%, while the high CA19–9 group had a survival rate of 35.5%. Thus, the survival rate was significantly poor in the high CA19–9 group (*p* < 0.001) (**Figure 1**). Concerning overall survival, the low CA19–9 group tended to have a higher survival rate than that of the high CA19–9 group, but the difference was not statistically significant (*p* = 0.173) (**Figure 2**).

## Discussion

The search for appropriate markers for the diagnosis and prediction prognosis of bladder cancer has been ongoing for a long time. Several studies have focused on serum biomarkers, which offer a relatively convenient, rapid, consistent, and low-complication testing method. Washino et al. found that CYFRA 21–1 was associated with high tumor stage, high tumor grade, disease progression, and disease-specific survival in advanced high-grade urothelial carcinoma of the bladder (7). Hegele et al. stated that although CEA and CA19–9 are not appropriate markers for the primary diagnosis of urothelial cancer, urothelial cancer should be suspected when elevated CA19–9 is observed, on the premise that gastrointestinal tract malignancy has been excluded (16). Wang et al. suggested that while CA19–9 has low sensitivity as a diagnostic marker in patients with bladder cancer, it is associated with overall survival and could be a powerful prognostic marker (13).

Among the markers described above, CA19–9 has been commonly used for diagnosis, prognosis evaluation, and recurrence surveillance in colon and pancreatic cancers (9, 17, 18). However, elevated levels of CA19–9 have also been observed in various other pathologies, such as liver disease, lung disease, gynecologic disease, and endocrine disorders, indicating that CA19–9 can be a useful biomarker in cancers beyond the gastrointestinal tract (19). Jian et al. reported that tumor occurrence and progression in bladder cancer are related to abnormal sialylation of glycans. Their finding reveals that CA19-9, same as sialyl Lewis A, has a possible molecular biological relationship with bladder cancer prognosis or recurrence (20).

There have been several specific studies conducted in various settings regarding the association between CA19–9 and bladder cancer. Margel et al. investigated CA19–9 levels in 91 patients who underwent radical cystectomy and reported that CA19–9 acts as an independent predictor of overall survival and disease-specific survival (21). Bazargani et al. measured pre-neoadjuvant chemotherapy CA19–9 levels in 337 patients with invasive bladder cancer and reported that those with higher CA19–9 levels had worse overall survival and recurrence-free survival (22). Nagao et al. measured urine CA19–9 levels and the gene phenotype of CA19-9 (Lewis gene/Secretor gene) in patients with bladder cancer, and revealed that patients with bladder cancer had higher urine CA19–9 levels compared to those of other urological disease groups in the Le/Se positive groups (23).

In the previous study, we have demonstrated that elevated CA19–9 levels in patients with UTUC serve as an indicator of high tumor burden and a poor prognostic predictor (10). In this study, we aimed to identify associations between elevated CA19–9 levels in patients with bladder cancer and tumor burden, prognosis, and survival. Our study focused on 564 patients with bladder cancer who underwent radical cystectomy and had available preoperative CA19–9 levels, allowing us to analyze clinical stage, pathologic stage, postoperative recurrence, and survival rates across all enrolled patients. In our findings, the clinical and pathologic T and N stages were significantly more advanced in the high CA19–9 group compared to those of the low CA19–9 group. This confirms that in patients who underwent radical cystectomy, higher CA19–9 levels are associated with more advanced cancer, ultimately suggesting a poor prognosis. In addition, CA19–9 level can contribute to guiding treatment decisions by serving as an indicator for determining treatment direction in patients at the borderline stage who may be considered for early radical cystectomy.

Elevated levels of CA 19−9 have repeatedly been linked to adverse outcomes in bladder cancer. Wang et al. reported that patients with advanced T stage or muscle invasive disease showed higher CA 19−9 levels among 144 bladder cancer patients (13). Our study confirms the association of CA 19−9 with T stage and extends it to lymphatic invasion, focusing exclusively on radical cystectomy patients. Ahmadi et al. reported that CA19–9 was associated with worse 3-year overall survival and recurrence-free survival in 186 cystectomy patients containing 7 patients with elevated CA19–9 levels (24). Our study involved a larger cohort of 564 cystectomy patients containing 50 patients with elevated CA19–9 levels. After adjusting for pathological T and N stage, CA 19−9 remained an independent predictor of recurrence (HR 1.646, p = 0.023) with longer follow-up period for 5 years. Collectively, these comparisons highlight that our study not only correlates with previous findings but also strengthens the prognostic value of CA 19–9 by adjusting tumor burden and providing the largest, extended follow-up cohort with radical cystectomy.

In addition, we considered the impact of the pathological stage on survival. To clarify the relationship between CA19–9 level and survival, we used multivariate Cox regression analysis with age, CA19–9 level, and pathological stage as covariates. In this analysis, the high CA19–9 group showed a significantly higher recurrence risk than the low CA19–9 group. This suggests that the CA19–9 level could be considered one of the effective markers for predicting recurrence after radical cystectomy.

Previous studies primarily demonstrated the association between CA19–9 and overall survival as well as disease-specific survival (13, 21). In our study, an analysis was conducted on a group of patients who underwent curative surgery, and both overall survival and recurrence-free survival were analyzed using Kaplan-Meier analysis. Although overall survival was not statistically significant, patients with bladder cancer in the high CA19–9 group tended to have a lower survival rate, which aligns with previous research findings. Additionally, in terms of recurrence-free survival, patients in the low CA19–9 group experienced more favorable outcomes. Based on this, it may be recommended that in the high CA19–9 group, more careful follow-up observation and surveillance for recurrence after curative surgical treatment should be performed.

This study has several limitations. First, the patient group included individuals who underwent neoadjuvant chemotherapy. In bladder cancer, preoperative chemotherapy may result in downstaging (25). Additionally, it has been proven that CA19–9 levels decrease when neoadjuvant chemotherapy is administered in other cancer types (26); thus, these factors may affect both CA19–9 levels and the prognosis evaluation of bladder cancer. However, when analyzing the patient group in this study, there was no significant difference in the proportion of patients who received neoadjuvant chemotherapy between the high and low CA19–9 groups. Therefore, the effects of downstaging and a decrease in CA19–9 levels after neoadjuvant chemotherapy were not considered to have a significant impact on analyzing various postoperative indicators.

Analysis according to variant histology was not conducted in this study. There are several subtypes within urothelial carcinoma of the bladder, such as micropapillary, plasmacytoid, and sarcomatoid. Particularly in cases such as micropapillary urothelial carcinoma, early radical cystectomy is often considered, and the treatment and prognosis appear to differ for each subtype (25, 27). Thus, research on CA19–9 level measurement and prognosis prediction depending on each subtype may be considered further.

Lastly, CA19–9 levels monitoring was not performed after surgery; therefore, the changes in CA19–9 levels after curative treatment were not evaluated. Despite these limitations, this study found that CA19–9 was significantly associated with clinical and pathologic T and N stages in patients with bladder cancer who underwent radical cystectomy, and CA19–9 could be used as a helpful prognostic biomarker in predicting recurrence and overall survival.

## Conclusion

In conclusion, a high CA19–9 level is associated with a high tumor burden and correlates with more advanced stages in both clinical T and N stages as well as pathologic T and N stages in patients with bladder cancer who underwent radical cystectomy. Moreover, a high CA19–9 level indicates worse recurrence-free survival after radical cystectomy.

## Data Availability

The raw data supporting the conclusions of this article will be made available by the authors, without undue reservation.
